# Lagrangian Function on the Finite State Space Statistical Bundle

**DOI:** 10.3390/e20020139

**Published:** 2018-02-22

**Authors:** Giovanni Pistone

**Affiliations:** De Castro Statistics, Collegio Carlo Alberto, 10122 Torino, Italy; giovanni.pistone@carloalberto.org

**Keywords:** Information Geometry, statistical bundle, Lagrangian function

## Abstract

The statistical bundle is the set of couples (Q,W) of a probability density *Q* and a random variable *W* such that ${𝔼}_{Q}\left[W\right]=0$. On a finite state space, we assume *Q* to be a probability density with respect to the uniform probability and give an affine atlas of charts such that the resulting manifold is a model for Information Geometry. Velocity and acceleration of a one-dimensional statistical model are computed in this set up. The Euler–Lagrange equations are derived from the Lagrange action integral. An example Lagrangian using minus the entropy as potential energy is briefly discussed.

## 1. Introduction

The set-up of classical Lagrangian Mechanics is a finite-dimensional Riemannian manifold. For example, see the monographs by V.I. Arnold ([1], Chapters III–IV), R. Abraham and J.E. Mardsen ([2], Chapter 3), J.E. Marsden and T.S. Ratiu ([3], Chapter 7). Classical Information geometry, as it was first defined in the monograph by S.-I. Amari and H. Nagaoka [4], views parametric statistical models as a manifold endowed with a dually-flat connection. In a recent paper, M. Leok and J. Zhang [5] have pointed out the natural relation between these two topics and have given a wide overview of the mathematical structures involved.

In the present paper, we take up the same research program with two further qualifications. First, we assume a non-parametric approach by considering the full set of positive probability functions on a finite set, as it was done, for example, in our review paper [6]. The discussion is restricted here to a finite state space to avoid difficult technical problems. Second, we consider a specific expression of the tangent space of the statistical manifold, which is a Hilbert bundle that we call a statistical bundle. Our aim is to emphasize the basic statistical intuition of the geometric quantities involved. Because of that, we chose to systematically use the language of non-parametric differential geometry as it is developed in the monography of S. Lang [7].

Herein, we use our version of Information Geometry; see the review paper [6]. Preliminary versions of this paper have been presented at the SigmaPhy2017 Conference held in Corfu, Greece, 10–14 July 2017, and at a seminar held at Collegio Carlo Alberto, Moncalieri, on 5 September 2017. In these early versions, we did not refer to Leok and Zhang’s work, which we were unaware of at that time.

In Section 2, we review the definition and properties of the statistical bundle, and of the affine atlas that endows it with both a manifold structure and a natural family of transports between the fibers. In Section 3, we develop the formalism of the tangent space of the statistical bundle and derive the expression of the velocity and the acceleration of a one-dimensional statistical model in the given affine atlas. The derivation of the Euler–Lagrange equations, together with a relevant example, is discussed in Section 4.

## 2. Statistical Bundle

We consider a finite sample space Ω, with #Ω=N. The probability simplex is Δ(Ω), and $\Delta^{\circ }\left(\Omega \right)$ is its interior. The uniform probability on Ω is denoted as μ, $\mu \left(x\right)=\frac{1}{N}$, x∈Ω. The *maximal exponential family* $\varepsilon \left(\mu \right)$ is the set of all strictly positive probability densities of (Ω,μ). The expected value of f:Ω→R with respect to the density $P\in \varepsilon \left(\mu \right)$ is denoted ${𝔼}_{P}\left[f\right]={𝔼}_{\mu }\left[fP\right]=\frac{1}{N}\sum_{x\in \Omega }f\left(x\right)P\left(x\right)$.

In [6,8,9], we made the case for the statistical bundle being the key structure of Information Geometry. The *statistical bundle* with base Ω is

$$S\varepsilon \left(\mu \right)=\left\{\left(Q,V\right)|Q\in \varepsilon \left(\mu \right),{𝔼}_{Q}\left[V\right]=0\right\}\;.$$

The statistical bundle is a semi-algebraic subset of $R^{2N}$; i.e., it is defined by algebraic equations and strict inequalities. It is trivially a real manifold. At each $Q\in \varepsilon \left(\mu \right)$, the fiber $S_{Q}\varepsilon \left(\mu \right)$ is endowed with the scalar product

$$\left(V_{1},V_{2}\right)\mapsto {\langle V_{1},V_{2}\rangle }_{Q}={𝔼}_{Q}\left[V_{1}V_{2}\right]={\mathrm{Cov}}_{Q}\left(V_{1},V_{2}\right)\;.$$

To this structure we add a special affine atlas of charts in order to show a structure of affine manifold, which is of interest in the statistical applications. The *exponential atlas* of the statistical manifold $S\varepsilon \left(\mu \right)$ is the collection of charts given for each $P\in \varepsilon \left(\mu \right)$ by

(1) $$s_{P}:S\varepsilon \left(\mu \right)∋\left(Q,V\right)\mapsto \left(s_{P}\left(Q\right),{}^{e}U_{Q}^{P}V\right)\in S_{P}\varepsilon \left(\mu \right)\times S_{P}\varepsilon \left(\mu \right)\;,$$

where (with a slight abuse of notation)

(2) $$s_{P}\left(Q\right)=\log\frac{Q}{P}-{𝔼}_{P}\left[\log\frac{Q}{P}\right],\;{}^{e}U_{Q}^{P}V=V-{𝔼}_{P}\left[V\right]\;.$$

As $s_{P}\left(P,V\right)=\left(0,V\right)$, we say that $s_{P}$ is the chart *centered at P*. If $s_{P}\left(Q\right)=U$, it is easy to derive the exponential form of *Q* as a density with respect to *P*; namely, $Q=e^{U-{𝔼}_{P}\left[\log\frac{Q}{P}\right]}\cdot P$. As ${𝔼}_{\mu }\left[Q\right]=1$, then $1={𝔼}_{p}\left[e^{U-{𝔼}_{P}\left[\log\frac{P}{Q}\right]}\right]={𝔼}_{p}\left[e^{U}\right]e^{-{𝔼}_{P}\left[\log\frac{P}{Q}\right]}$, so that the *cumulant function* $K_{P}$ is defined on $S_{P}\varepsilon \left(\mu \right)$ by

$$K_{p}\left(U\right)=\log{𝔼}_{P}\left[e^{U}\right]={𝔼}_{P}\left[\log\frac{P}{Q}\right]=D\left(P\;∥Q\right)\;;$$

that is, $K_{P}\left(V\right)$ is the expression in the chart at *P* of Kullback–Leibler divergence of $Q\mapsto D\left(P\;∥Q\right)$, and we can write

$$Q=e^{U-K_{P}\left(U\right)}\times P=e_{P}\left(U\right)\;.$$

The *patch centered at P* is

$$s_{P}^{-1}=e_{P}:{\left(S_{P}\varepsilon \left(\mu \right)\right)}^{2}∋\left(U,W\right)\mapsto \left(e_{P}\left(U\right),{}^{e}U_{P}^{e_{P}\left(U\right)}W\right)\in S\varepsilon \left(\mu \right)\;.$$

In statistical terms, the random variable $\log\left(Q/P\right)$ is the relative point-wise information about *Q* relative to the reference *P*, while $s_{P}\left(Q\right)$ is the deviation from its mean value at *P*. The expression of the other divergence in the chart centered at *P* is

$$D\left(Q\;∥P\right)={𝔼}_{Q}\left[\log\frac{Q}{P}\right]={𝔼}_{Q}\left[U-K_{P}\left(U\right)\right]={𝔼}_{Q}\left[U\right]-K_{P}\left(U\right)\;.$$

The equation above shows that the two divergences are convex conjugate functions in the proper charts; see [10].

The transition maps of the exponential atlas in Equations (1) and (2) are

$$\begin{array}{c} s_{P_{2}}\circ e_{P_{1}}\left(U,W\right)=s_{P_{2}}\left(e_{P_{1}}\left(U\right),{}^{e}U_{P}^{e_{1}P\left(U\right)}W\right)=s_{P_{2}}\left(e^{U-K_{P_{1}}\left(U\right)}\times P_{1},W-{𝔼}_{e_{P_{1}}\left(U\right)}\left[W\right]\right)=\\ \left(U-K_{P_{1}}\left(U\right)+\log\frac{P_{1}}{P_{2}}-{𝔼}_{P_{2}}\left[U-K_{P_{1}}\left(U\right)+\log\frac{P_{1}}{P_{2}}\right],W-{𝔼}_{e_{P_{1}}\left(U\right)}\left[W\right]-{𝔼}_{P_{2}}\left[W-{𝔼}_{e_{P_{1}}\left(U\right)}\left[W\right]\right]\right)=\\ \left({}^{e}U_{P_{1}}^{P_{2}}U+s_{P_{2}}\left(P_{1}\right),{}^{e}U_{P_{1}}^{P_{2}}W\right)\;, \end{array}$$

so that the exponential atlas is indeed affine. Notice that the linear part is ${}^{e}U_{P_{1}}^{P_{2}}$.

## 3. The Tangent Space of the Statistical Bundle

Let us compute the expression of the velocity at time *t* of a smooth curve $t\mapsto \gamma \left(t\right)=\left(Q\left(t\right),W\left(t\right)\right)\in S\varepsilon \left(\mu \right)$ in the chart centered at *P*. The expression of the curve is

$$\gamma_{P}\left(t\right)=s_{P}\left(\gamma \left(t\right)\right)=\left(s_{P}\left(Q\left(t\right)\right),{}^{e}U_{Q(t)}^{P}W\left(t\right)\right)\;,$$

and hence we have, by denoting the derivative in $R^{N}$ by the dot,

(3) $$\frac{d}{dt}s_{P}\left(Q\left(t\right)\right)=\frac{d}{dt}\left(\log\frac{Q(t)}{P}-{𝔼}_{P}\left[\log\frac{Q(t)}{P}\right]\right)=\frac{\dot{Q}\left(t\right)}{Q(t)}-{𝔼}_{P}\left[\frac{\dot{Q}\left(t\right)}{Q(t)}\right]={}^{e}U_{Q(t)}^{P}\frac{\dot{Q}\left(t\right)}{Q(t)}\;,$$

and

(4) $$\frac{d}{dt}{}^{e}U_{Q(t)}^{P}W\left(t\right)=\frac{d}{dt}\left(W\left(t\right)-{𝔼}_{P}\left[W(t)\right]\right)=\dot{W}\left(t\right)-{𝔼}_{P}\left[\dot{W}\left(t\right)\right]={}^{e}U_{Q(t)}^{P}\left(\dot{W}\left(t\right)-{𝔼}_{Q(t)}\left[\dot{W}\left(t\right)\right]\right)\;.$$

If we define the *velocity* of $t\mapsto Q\left(t\right)=e^{U\left(t\right)-K_{p}\left(U\left(t\right)\right)}\times P$ to be

$$\overset{⋆}{Q}\left(t\right)=\frac{\dot{Q}\left(t\right)}{Q(t)}=\frac{d}{dt}\log Q\left(t\right)=\dot{U}\left(t\right)-dK_{P}\left(U\left(t\right)\right)\left[\dot{U}\left(t\right)\right]\in S_{Q(t)}\varepsilon \left(\mu \right)\;,$$

then $t\mapsto (Q\left(t\right),\overset{⋆}{Q}\left(t\right))$ is a curve in the statistical bundle whose expression in the chart centered at *P* is $t\mapsto (U\left(t\right),\dot{U}\left(t\right))$. The velocity as defined above is nothing else as the *score function* of the one-dimensional statistical model; see e.g., the textbook by B. Efron and T. Haste (Section 4.2, [11]). The variance of the score function (i.e., the squared norm of $\overset{⋆}{Q}\left(t\right)$ in $S_{Q(t)}\varepsilon \left(\mu \right)$) is classically known as *Fisher information* at *t*.

We define the *second statistical bundle* to be

$$S^{2}\varepsilon \left(\mu \right)=\left\{\left(Q,W,X,Y\right)|\left(Q,W\right)\in S\varepsilon \left(\mu \right),X,Y\in S_{Q}\varepsilon \left(\mu \right)\right\}\;,$$

with charts

$$s_{P}\left(Q,V,X,Y\right)=\left(s_{P}\left(Q,V\right),{}^{e}U_{Q}^{P}X,{}^{e}U_{Q}^{P}Y\right)\;,$$

we can identify the second bundle with the tangent space of the first bundle as follows.

For each curve t↦γ(t)=(Q(t),W(t)) in the statistical bundle, define its *velocity at t* to be

$$\overset{⋆}{\gamma }\left(t\right)=\left(Q\left(t\right),W\left(t\right),\overset{⋆}{Q}\left(t\right),\dot{W}\left(t\right)-{𝔼}_{Q(t)}\left[\dot{W}\left(t\right)\right]\right)\;,$$

because $t\mapsto \overset{⋆}{\gamma }\left(t\right)$ is a curve in the second statistical bundle, and its expression in the chart at *P* has the last two components equal to the values given in Equations (3) and (4).

In particular, consider the a curve $t\mapsto \chi \left(t\right)=(Q\left(t\right),\overset{⋆}{Q}\left(t\right))$. The velocity is

$$\overset{⋆}{\chi }\left(t\right)=\left(Q\left(t\right),\overset{⋆}{Q}\left(t\right),\overset{⋆}{Q}\left(t\right),\overset{**}{Q}\left(t\right)\right)\;,$$

where the *acceleration* $\overset{**}{Q}\left(t\right)$ is

(5) $$\overset{**}{Q}\left(t\right)=\frac{d}{dt}\frac{\dot{Q}\left(t\right)}{Q(t)}-{𝔼}_{Q(t)}\left[\frac{d}{dt}\frac{\dot{Q}\left(t\right)}{Q(t)}\right]=\frac{\ddot{Q}\left(t\right)}{Q(t)}-\left(\overset{⋆}{Q}{\left(t\right)}^{2}-{𝔼}_{Q(t)}\left[\overset{⋆}{Q}{\left(t\right)}^{2}\right]\right)\;$$

It should be noted that the acceleration has been defined without explicitly mentioning the relevant connection. In fact, the connection here is implicitly defined by the transports ${}^{e}U_{P}^{Q}$, which is unusual in Differential Geometry, but is quite natural from the probabilistic point of view; see P. Gibilisco and G. Pistone [12]. We shall see below that the non-parametric approach to Information Geometry allows the definition of a dual transport, hence a dual connection as it was in [4]. Because of that, we could have defined other types of acceleration together with the one we have defined. Namely, we could consider an *exponential acceleration* ${}^{e}D^{2}Q\left(t\right)=\overset{**}{Q}\left(t\right)$, a *mixture acceleration* ${}^{m}D^{2}Q\left(t\right)=\ddot{Q}\left(t\right)/Q\left(t\right)$, and a *Riemannian acceleration*

(6) $${}^{0}D^{2}Q\left(t\right)=\frac{1}{2}\left({}^{e}D^{2}Q\left(t\right)+{}^{m}D^{2}Q\left(t\right)\right)=\frac{\ddot{Q}\left(t\right)}{Q(t)}-\frac{1}{2}\left({\left(\frac{\dot{Q}\left(t\right)}{Q(t)}\right)}^{2}-{𝔼}_{Q(t)}\left[{\left(\frac{\dot{Q}\left(t\right)}{Q(t)}\right)}^{2}\right]\right)\;,$$

each acceleration being associated with a specific connection; see the review paper [6]. We do not further discuss the different second-order geometries associated with the statistical bundle in this paper.

**Example** **1** (Boltzmann–Gibbs). Let us compare the formalism we have introduced above with standard computations in Statistical Physics. The *Boltzmann–Gibbs distribution* gives to point x∈Ω the probability $e^{-(1/\theta )H(x)}/Z\left(\theta \right)$, with $Z\left(\theta \right)=\sum_{x\in \Omega }e^{-(1/\theta )H(x)}$ and θ>0, see Landau and Lifshitz ([13], Chapter 3). As a curve in $\varepsilon \left(\mu \right)$, it is $Q\left(\theta \right)=Ne^{-(1/\theta )H}/Z\left(\theta \right)$ because of the reference to the uniform probability. The velocity defined above becomes in this case $\overset{⋆}{Q}\left(\theta \right)=\theta^{-2}\left(H-{𝔼}_{\theta }\left[H\right]\right)$, while the acceleration of Equation (5) is $\overset{**}{Q}\left(\theta \right)=-\theta^{-3}\left(H-{𝔼}_{\theta }\left[H\right]\right)$. Notice that we have the equation $\theta \overset{**}{Q}\left(\theta \right)+\overset{⋆}{Q}\left(\theta \right)=0$.

Following the original construction of Amari’s Information Geometry [4], we have defined on the statistical bundle a manifold structure which is both an affine and a Riemannian manifold. The base manifold $\varepsilon \left(\mu \right)$ is actually a Hessian manifold with respect to any of the convex functions $K_{p}\left(U\right)=\log{𝔼}_{p}\left[e^{U}\right]$, $U\in S_{p}\varepsilon \left(\mu \right)$ (see [14]). Many computations are actually performed using the Hessian structure. The following equations are easily checked and frequently used:

(7) $$\begin{array}{cc} {𝔼}_{e_{P}\left(U\right)}\left[H\right] & =dK_{P}\left(U\right)\left[H\right]\;; \end{array}$$

(8) $$\begin{array}{cc} {}^{e}U_{P}^{e_{P}\left(U\right)}H & =H-dK_{P}\left(U\right)\left[H\right]\;; \end{array}$$

(9) $$\begin{array}{cc} d^{2}K_{P}\left(U\right)\left[H_{1},H_{2}\right] & ={\langle {}^{e}U_{P}^{e_{P}\left(U\right)}H_{1},{}^{e}U_{P}^{e_{P}\left(U\right)}H_{2}\rangle }_{e_{P}\left(U\right)}\;; \end{array}$$

(10) $$\begin{array}{cc} d^{3}K_{p}\left(U\right)\left[H_{1},H_{2},H_{3}\right] & ={𝔼}_{e_{P}\left(U\right)}\left[{}^{e}U_{P}^{e_{P}\left(U\right)}H_{1}\times {}^{e}U_{P}^{e_{P}\left(U\right)}H_{2}\times {}^{e}U_{P}^{e_{P}\left(U\right)}H_{3}\right]\;. \end{array}$$

We have defined a centering operation that can be thought of as a *transport* among fibers,

$${}^{e}U_{P}^{Q}:S_{p}\varepsilon \left(\mu \right)\to S_{q}\varepsilon \left(\mu \right)\;,$$

whose adjoint is ${}^{m}U_{q}^{p}V=\frac{q}{p}V$. In fact, is the adjoint of ${}^{e}U_{p}^{q}$,

$${\langle {}^{e}U_{P}^{Q}U,V\rangle }_{Q}={𝔼}_{Q}\left[(U-{𝔼}_{Q}\left[U\right])V\right]={𝔼}_{Q}\left[UV\right]={𝔼}_{P}\left[U\left(\frac{Q}{P}V\right)\right]={\langle U,{}^{m}U_{Q}^{P}V\rangle }_{P}$$

Moreover, iff $U,V\in S_{P}\varepsilon \left(\mu \right)$, then

$${\langle {}^{e}U_{P}^{Q}U,{}^{m}U_{P}^{Q}V\rangle }_{Q}={\langle {}^{e}U_{Q}^{P}{}^{e}U_{P}^{Q}U,V\rangle }_{P}={\langle U,V\rangle }_{P}\;.$$

**Example** **2** (Entropy flow). This example is taken from [8]. In the scalar field $\varepsilon \left(Q\right)=-{𝔼}_{Q}\left[\log Q\right]$, there is no dependence on the fiber. If $t\mapsto Q\left(t\right)=e^{V\left(t\right)-K_{P}\left(V\left(t\right)\right)}\cdot P$ is a smooth curve in $\varepsilon \left(\mu \right)$ expressed in the chart centered at *P*, then we can write

(11) $$\begin{array}{cc} ℋ\left(Q(t)\right)=-{𝔼}_{Q(t)}\left[V\left(t\right)-K_{P}\left(V\left(t\right)\right)+\log P\right]= & \\ & K_{P}\left(V\left(t\right)\right)-{𝔼}_{Q(t)}\left[V\left(t\right)+\log P+ℋ\left(P\right)\right]+ℋ\left(P\right)=\\ & K_{P}\left(V\left(t\right)\right)-dK_{P}\left(V\left(t\right)\right)\left[V\left(t\right)+\log P+ℋ\left(P\right)\right]+ℋ\left(P\right)\;, \end{array}$$

where the argument of the last expectation belongs to the fiber $S_{P}\varepsilon \left(\mu \right)$ and we have expressed the expected value as a derivative by using Equation (7).

Again using Equations (7) and (9), we compute the derivative of the entropy along the given curve as

$$\begin{array}{cc} \frac{d}{dt}ℋ\left(Q(t)\right) & =\frac{d}{dt}K_{P}\left(V\left(t\right)\right)-\frac{d}{dt}dK_{P}\left(V\left(t\right)\right)\left[V\left(t\right)+\log P+ℋ\left(P\right)\right]=\\ & dK_{P}\left(V\left(t\right)\right)\left[\dot{V}\left(t\right)\right]-d^{2}K_{P}\left(V\left(t\right)\right)\left[V\left(t\right)+\log P+ℋ\left(P\right),\dot{V}\left(t\right)\right]-dK_{P}\left(V\left(t\right)\right)\left[\dot{V}\left(t\right)\right]=\\ & -{𝔼}_{Q(t)}\left[{}^{e}U_{P}^{Q(t)}\left(V\left(t\right)+\log P\right){}^{e}U_{P}^{Q(t)}\dot{V}\left(t\right)\right]\;. \end{array}$$

We use now the equations

$$V\left(t\right)+\log P=\log Q\left(t\right)+K_{P}\left(V\left(t\right)\right)\;,\;{}^{e}U_{P}^{Q(t)}\left(\log Q\left(t\right)+K_{P}\left(V\left(t\right)\right)\right)=\log Q\left(t\right)+ℋ\left(Q(t)\right)\;,$$

and ${}^{e}U_{P}^{Q(t)}\dot{V}\left(t\right)=\overset{⋆}{Q}\left(t\right)$ to obtain

$$\frac{d}{dt}ℋ\left(Q(t)\right)=-{\langle \log Q\left(t\right)+ℋ\left(Q(t)\right),\overset{⋆}{Q}\left(t\right)\rangle }_{Q(t)}\;.$$

We have identified the gradient of the entropy in the statistical bundle,

(12) $$\mathrm{grad}ℋ\left(Q\right)=-\left(\log Q+ℋ\left(Q\right)\right)\;.$$

Notice that the previous computation could have been done using the exponential family $Q\left(t\right)=e_{P}\left(tV\right)$. See the computation of the gradient flow in [8].

In the next section, we extend the computation illustrated in the example above to scalar fields on the statistical bundle.

## 4. Lagrangian Function

A *Lagrangian function* is a smooth scalar field on the statistical bundle

$$L:S\varepsilon \left(\mu \right)∋\left(Q,W\right)\mapsto L\left(Q,W\right)\in R\;.$$

At each fixed density $Q\in \varepsilon \left(\mu \right)$, the partial mapping

(13) $$S_{Q}\varepsilon \left(\mu \right)∋W\mapsto L\left(Q,W\right)$$

is defined on the vector space $S_{q}\varepsilon \left(\mu \right)$; hence, we can use the ordinary derivative, which in this case is called the *fiber derivative*,

(14) $$d_{2}L\left(Q,W\right)\left[H_{2}\right]={\left.\frac{d}{dt}L\left(Q,W+tH_{2}\right)\right|}_{t=0},\;H_{2}\in S_{Q}\varepsilon \left(\mu \right)\;.$$

**Example** **3** (Running Example 1). If

(15) $$L\left(Q,W\right)=\frac{1}{2}{\langle W,W\rangle }_{Q}+\kappa ℋ\left(Q\right)\;,\;\kappa \geq o\;,$$

then $d_{2}L\left(Q,W\right)\left[H_{2}\right]={\langle W,H_{2}\rangle }_{Q}$. The example is suggested by the form of the classical Lagrangian function in mechanics, where the first term is the kinetic energy and $-\kappa \varepsilon \left(Q\right)$ is the potential energy.

As the statistical bundle $S\varepsilon \left(\mu \right)$ is non-trivial, the computation of the partial derivative of the Lagrangian with respect to the first variable requires some care. We want to compute the expression of the total derivative in a chart of the affine atlas defined in Equations (1) and (2).

Let t↦γ(t)=(Q(t),W(t)) be a smooth curve in the statistical bundle. In the chart centered at *P*, we have

$$Q\left(t\right)=e^{U\left(t\right)-K_{P}\left(U\left(t\right)\right)}\times P=e_{P}\left(U\left(t\right)\right),\;W\left(t\right)={}^{e}U_{P}^{e_{P}\left(U\left(t\right)\right)}V\left(t\right)\;,$$

with $t\mapsto \gamma_{P}\left(t\right)=\left(U\left(t\right),V\left(t\right)\right)$ being a smooth curve in ${\left(S_{P}\varepsilon \left(\mu \right)\right)}^{2}$. Let us compute the velocity of variation of the Lagrangian *L* along the curve γ.

$$\frac{d}{dt}L\left(\gamma \left(t\right)\right)=\frac{d}{dt}L\left(Q\left(t\right),W\left(t\right)\right)=\frac{d}{dt}L\left(e_{P}\left(U\left(t\right)\right),{}^{e}U_{P}^{e_{P}\left(U\left(t\right)\right)}V\left(t\right)\right)=\frac{d}{dt}L_{P}\left(U\left(t\right),V\left(t\right)\right)\;,$$

with $L_{P}\left(U,V\right)=L\left(e_{P}\left(U\right),{}^{e}U_{P}^{e_{P}\left(U\right)}V\right)$. It follows that

(16) $$\frac{d}{dt}L\left(Q\left(t\right),W\left(t\right)\right)=d_{1}L_{P}\left(U\left(t\right),V\left(t\right)\right)\left[\dot{U}\left(t\right)\right]+d_{2}L_{P}\left(U\left(t\right),V\left(t\right)\right)\left[\dot{V}\left(t\right)\right]\;.$$

If we write $Q=e_{P}\left(U\right)$ and $W={}^{e}U_{P}^{e_{P}\left(U\right)}V$, then we have

(17) $$d_{2}L_{P}\left(U,V\right)\left[H_{2}\right]={\left.\frac{d}{dt}L_{P}\left(U,V+tH_{2}\right)\right|}_{t=0}={\left.\frac{d}{dt}L\left(Q,W+t{}^{e}U_{P}^{Q}H_{2}\right)\right|}_{t=0}=d_{2}L\left(Q,W\right)\left[{}^{e}U_{P}^{Q}H_{2}\right]\;,$$

where $d_{2}L$ is the fiber derivative of *L*. As $\dot{U}\left(t\right)={}^{e}U_{Q(t)}^{P}\overset{⋆}{Q}\left(t\right)$ and ${}^{e}U_{P}^{e_{P}\left(U\left(t\right)\right)}\dot{V}\left(t\right)=\overset{⋆}{W}\left(t\right)$, it follows from Equations (16) and (17) that

$$\frac{d}{dt}L\left(Q\left(t\right),W\left(t\right)\right)=d_{1}L_{P}\left(U\left(t\right),V\left(t\right)\right)\left[{}^{e}U_{Q(t)}^{P}\overset{⋆}{Q}\left(t\right)\right]+d_{2}L\left(Q\left(t\right),W\left(t\right)\right)\left[\overset{⋆}{W}\left(t\right)\right]\;.$$

In the equation above, the first term on the RHS does not depend on *P* because the LHS and the second term of the RHS do not depend on *P*. Hence, we define the first partial derivative of the Lagrangian function to be

(18) $$d_{1}\left(Q,W\right)\left[H_{1}\right]=d_{1}L_{P}\left(U,V\right)\left[{}^{e}U_{e_{P}\left(U\right)}^{P}H_{1}\right]\;,\;H_{1}\in S_{Q}\varepsilon \left(\mu \right)\;,$$

so that the derivative of *L* along γ becomes

(19) $$\frac{d}{dt}L\left(Q\left(t\right),W\left(t\right)\right)=d_{1}L\left(Q\left(t\right),W\left(t\right)\right)\left[\overset{⋆}{Q}\left(t\right)\right]+d_{2}L\left(Q\left(t\right),W\left(t\right)\right)\left[\overset{⋆}{W}\left(t\right)\right]\;.$$

In particular, if $W\left(t\right)=\overset{⋆}{Q}\left(t\right)$, then

$$\frac{d}{dt}L\left(Q\left(t\right),\overset{⋆}{Q}\left(t\right)\right)=d_{1}L\left(Q\left(t\right),\overset{⋆}{Q}\left(t\right)\right)\left[\overset{⋆}{Q}\left(t\right)\right]+d_{2}L\left(Q\left(t\right),\overset{⋆}{Q}\left(t\right)\right)\left[\overset{**}{Q}\left(t\right)\right]\;,$$

see Equation (5).

**Example** **4** (Running Example 2). With the Lagrangian of Equation (15), we have

$$\begin{array}{c} L_{P}\left(U,V\right)=\frac{1}{2}{\langle {}^{e}U_{P}^{e_{P}\left(U\right)}V,{}^{e}U_{P}^{e_{P}\left(U\right)}V\rangle }_{e_{P}\left(U\right)}-\kappa {𝔼}_{e_{P}\left(U\right)}\left[U-K_{P}\left(U\right)+\log P\right]=\\ \frac{1}{2}d^{2}K_{P}\left(U\right)\left[V,V\right]+\kappa \left(K_{P}\left(U\right)-dK_{P}\left(U\right)\left[U+\log P+ℋ\left(P\right)\right]+ℋ\left(P\right)\right)\;, \end{array}$$

see Equations (9) and (11). The first partial derivative is

$$\begin{array}{cc} d_{1}L_{P}\left(U,V\right)\left[H_{1}\right]= & \\ & \frac{1}{2}d^{3}K_{P}\left(U\right)\left[V,V,H_{1}\right]+\kappa \left(dK_{P}\left(U\right)\left[H_{1}\right]-d^{2}K_{P}\left(U\right)\left[U+\log P+ℋ\left(P\right),H_{1}\right]-dK_{P}\left(U\right)\left[H_{1}\right]\right)=\\ & \frac{1}{2}d^{3}K_{P}\left(U\right)\left[V,V,H_{1}\right]-\kappa d^{2}K_{P}\left(U\right)\left[U+\log P+ℋ\left(P\right),H_{1}\right]=\\ & \frac{1}{2}{𝔼}_{Q}\left[W^{2}{}^{e}U_{P}^{e_{P}\left(U\right)}H_{1}\right]-\kappa {𝔼}_{Q}\left[\left(\log Q+ℋ\left(Q\right)\right){}^{e}U_{P}^{e_{P}\left(U\right)}H_{1}\right]=\\ & {𝔼}_{Q}\left[\left(\frac{1}{2}\left(W^{2}-{𝔼}_{Q}\left[W^{2}\right]\right)-\kappa \left(\log Q+ℋ\left(Q\right)\right)\right){}^{e}U_{P}^{e_{P}\left(U\right)}H_{1}\right]\;, \end{array}$$

where we have used Equations (9) and (10) together with ${}^{e}U_{P}^{e_{P}\left(U\right)}\left(U+\log P+ℋ\left(P\right)\right)=\log Q+ℋ\left(Q\right)$.

We have found that

(20) $$d_{1}L\left(Q,W\right)\left[H_{1}\right]={\langle \frac{1}{2}\left(W^{2}-{𝔼}_{Q}\left[W^{2}\right]\right)-\kappa \left(\log Q+ℋ\left(Q\right)\right),H_{1}\rangle }_{Q}\;,\;H_{1}\in S_{Q}\varepsilon \left(\mu \right)\;,$$

and also

$$d_{1}L\left(Q\left(t\right),\overset{⋆}{Q}\left(t\right)\right)\left[\overset{⋆}{Q}\left(t\right)\right]={\langle \frac{1}{2}\left({\overset{⋆}{Q}\left(t\right)}^{2}-{𝔼}_{Q}\left[{\overset{⋆}{Q}\left(t\right)}^{2}\right]\right)-\kappa \left(\log Q+ℋ\left(Q\right)\right),\overset{⋆}{Q}\left(t\right)\rangle }_{Q}\;.$$

Using the fiber derivative computed in the first part of the running example, we find

$$\frac{d}{dt}L\left(Q\left(t\right),\overset{⋆}{Q}\left(t\right)\right)={\langle \frac{1}{2}\left({\overset{⋆}{Q}\left(t\right)}^{2}-{𝔼}_{Q}\left[{\overset{⋆}{Q}\left(t\right)}^{2}\right]\right)-\kappa \left(\log Q+ℋ\left(Q\right)\right),\overset{⋆}{Q}\left(t\right)\rangle }_{Q}+{\langle \overset{⋆}{Q}\left(t\right),\overset{**}{Q}\left(t\right)\rangle }_{Q}\;.$$

Notice that Equation (12) shows that one of the terms in the equations above is $\mathrm{grad}ℋ\left(Q\right)$.

## 5. Action Integral

If [0,1]∋t↦Q(t) is a smooth curve in the exponential manifold, then the *action integral*

$$A\left(Q\right)=\int_{0}^{1}L\left(Q\left(t\right),\overset{⋆}{Q}\left(t\right)\right)\;dt$$

is well defined. We consider the expression of *Q* in the chart centered at *P*, $Q\left(t\right)=e^{U\left(t\right)-K_{P}\left(U\left(t\right)\right)}\times P$.

Given $\varphi \in C^{1}\left(\left[0,1\right]\right)$ with φ(0)=φ(1)=0, for each δ∈R and $H\in S_{P}\varepsilon \left(\mu \right)$, we define the perturbed curve

$$Q_{\delta }\left(t\right)=e^{\left(U\left(t\right)+\delta \varphi \left(t\right)H\right)-K_{P}\left(U\left(t\right)+\delta \varphi \left(t\right)H\right)}\times P\;.$$

We have $Q_{\delta }\left(0\right)=Q\left(0\right)$, $Q_{\delta }\left(1\right)=Q\left(1\right)$, and

$${\overset{⋆}{Q}}_{\delta }\left(t\right)=\dot{U}\left(t\right)+\delta \dot{\varphi }\left(t\right)H-{𝔼}_{Q_{\delta }\left(t\right)}\left[(\dot{U}\left(t\right)+\delta \dot{\varphi }\left(t\right))H\right]\;,$$

whose expression in the chart centered at *P* is $\dot{U}\left(t\right)+\delta \dot{\varphi }\left(t\right)H$.

Let us consider the variation in δ of the action integral. We apply Equation (19) applied to the smooth curve in $S\varepsilon \left(\mu \right)$ given by

$$\delta \mapsto (Q_{\delta }\left(t\right),{\overset{⋆}{Q}}_{\delta }\left(t\right))\;,$$

where *t* is fixed. As

$$\frac{d}{d\delta }\log Q_{\delta }\left(t\right)=\frac{d}{d\delta }\left(U(t)+\delta \varphi (t)H\right)-{𝔼}_{Q_{\delta }\left(t\right)}\left[\frac{d}{d\delta }\left(U(t)+\delta \varphi (t)H\right)\right]=\varphi \left(t\right)\left(H-{𝔼}_{Q_{\delta }\left(t\right)}\left[H\right]\right)$$

and

$${}^{e}U_{P}^{Q_{\delta }\left(t\right)}\frac{d}{d\delta }\left(\dot{U}\left(t\right)+\delta \dot{\varphi }\left(t\right)H\right)=\dot{\varphi }\left(t\right)\left(H-{𝔼}_{Q_{\delta }\left(t\right)}\left[H\right]\right)\;,$$

we obtain

$$\begin{array}{c} \frac{d}{d\delta }A\left(Q_{\delta }\right)=\int_{0}^{1}\frac{d}{d\delta }L\left(Q_{\delta }\left(t\right),{\overset{⋆}{Q}}_{\delta }\left(t\right)\right)\;dt=\\ \int_{0}^{1}\left(\varphi \left(t\right)d_{1}L\left(Q_{\delta }\left(t\right),{\overset{⋆}{Q}}_{\delta }\left(t\right)\right)\left[H-{𝔼}_{Q_{\delta }\left(t\right)}\left[H\right]\right]+\dot{\varphi }\left(t\right)d_{2}L\left(Q_{\delta }\left(t\right),{\overset{⋆}{Q}}_{\delta }\left(t\right)\right)\left[H-{𝔼}_{Q_{\delta }\left(t\right)}\left[H\right]\right]\right)\;dt=\\ \int_{0}^{1}\varphi \left(t\right)\left(d_{1}L\left(Q_{\delta }\left(t\right),{\overset{⋆}{Q}}_{\delta }\left(t\right)\right)\left[H-{𝔼}_{Q_{\delta }\left(t\right)}\left[H\right]\right]-\frac{d}{dt}d_{2}L\left(Q_{\delta }\left(t\right),{\overset{⋆}{Q}}_{\delta }\left(t\right)\right)\left[H-{𝔼}_{Q_{\delta }\left(t\right)}\left[H\right]\right]\right)\;dt\;. \end{array}$$

If t↦Q(t) is a critical curve of the action integral, then ${\left.\frac{d}{d\delta }A\left(Q_{\delta }\right)\right|}_{\delta =0}=0$; hence, for all φ and *H*, we have

(21) $$\int_{0}^{1}\varphi \left(t\right)\left(d_{1}L\left(Q\left(t\right),\overset{⋆}{Q}\left(t\right)\right)\left[H-{𝔼}_{Q(t)}\left[H\right]\right]-\frac{d}{dt}d_{2}L\left(Q\left(t\right),\overset{⋆}{Q}\left(t\right)\right)\left[H-{𝔼}_{Q(t)}\left[H\right]\right]\right)\;dt=0\;.$$

This in turn implies that for each t∈[0,1] and $H\in S_{Q(t)}\varepsilon \left(\mu \right)$, the Euler–Lagrange equation holds:

(22) $$d_{1}L\left(Q\left(t\right),\overset{⋆}{Q}\left(t\right)\right)\left[H\right]-\frac{d}{dt}d_{2}L\left(Q\left(t\right),\overset{⋆}{Q}\left(t\right)\right)\left[H\right]=0\;.$$

**Example** **5** (Running Example 3). For the Lagrangian of Equation (15), we can use Equation (20) in the form

$$\begin{array}{c} d_{1}L\left(Q\left(t\right),\overset{⋆}{Q}\left(t\right)\right)\left[H-{𝔼}_{Q(t)}\left[H\right]\right]=\\ {\langle \frac{1}{2}\left(\overset{⋆}{Q}{\left(t\right)}^{2}-{𝔼}_{Q(t)}\left[\overset{⋆}{Q}{\left(t\right)}^{2}\right]\right)-\kappa \left(\log\left(Q(t)\right)+ℋ\left(Q(t)\right)\right),H-{𝔼}_{Q(t)}\left[H\right]\rangle }_{Q(t)}\;, \end{array}$$

with $H\in S_{P}\varepsilon \left(\mu \right)$. For the other term, we have

$$d_{2}L\left(Q\left(t\right),\overset{⋆}{Q}\left(t\right)\right)\left[H-{𝔼}_{Q(t)}\left[H\right]\right]={\langle \overset{⋆}{Q}\left(t\right),H-{𝔼}_{Q(t)}\left[H\right]\rangle }_{Q(t)}=d^{2}K_{P}\left(U\left(t\right)\right)\left[\dot{U}\left(t\right),H\right]\;,$$

whose derivative is

$$\begin{array}{c} \frac{d}{dt}\;d^{2}K_{P}\left(U\left(t\right)\right)\left[\dot{U}\left(t\right),HR\right]=d^{3}K_{P}\left(U\left(t\right)\right)\left[\dot{U}\left(t\right),\dot{U}\left(t\right),H\right]+d^{2}K_{P}\left(U\left(t\right)\right)\left[\ddot{U}\left(t\right),H\right]=\\ {𝔼}_{Q(t)}\left[\overset{⋆}{Q}{\left(t\right)}^{2}\left(H-{𝔼}_{Q(t)}\left[H\right]\right)\right]+{𝔼}_{Q(t)}\left[\overset{**}{Q}\left(t\right)\left(H-{𝔼}_{Q(t)}\left[H\right]\right)\right]=\\ {𝔼}_{Q(t)}\left[\left(\overset{⋆}{Q}{\left(t\right)}^{2}-{𝔼}_{Q(t)}\left[\overset{⋆}{Q}{\left(t\right)}^{2}\right]\right)\left(H-{𝔼}_{Q(t)}\left[H\right]\right)\right]+{𝔼}_{Q(t)}\left[\overset{**}{Q}\left(t\right)\left(H-{𝔼}_{Q(t)}\left[H\right]\right)\right]\;. \end{array}$$

Dropping the generic *H*, the Euler–Lagrange equation becomes

$$\overset{**}{Q}\left(t\right)+\left(\overset{⋆}{Q}{\left(t\right)}^{2}-{𝔼}_{Q(t)}\left[\overset{⋆}{Q}{\left(t\right)}^{2}\right]\right)=\frac{1}{2}\left(\overset{⋆}{Q}{\left(t\right)}^{2}-{𝔼}_{Q(t)}\left[\overset{⋆}{Q}{\left(t\right)}^{2}\right]\right)-\kappa \left(\log\left(Q(t)\right)+ℋ\left(Q(t)\right)\right)\;;$$

that is,

$$\overset{**}{Q}\left(t\right)+\frac{1}{2}\left(\overset{⋆}{Q}{\left(t\right)}^{2}-{𝔼}_{Q(t)}\left[\overset{⋆}{Q}{\left(t\right)}^{2}\right]\right)=-\kappa \left(\log\left(Q(t)\right)+ℋ\left(Q(t)\right)\right)\;.$$

The equation above has been derived using the exponential affine geometry of the statistical bundle and involves $\overset{**}{Q}\left(t\right)$. However, by using Equations (5), (6), and (12), we find the equivalent form

$${}^{0}D^{2}Q\left(t\right)=\kappa \mathrm{grad}ℋ\left(Q(t)\right)\;.$$

## 6. Discussion

We have shown that the research program consisting of applying concepts taken from Classical Mechanics to Statistics makes sense, even if no practical application has been produced in this paper. Some simple examples have been discussed in order to show clearly that the language from classical mechanics is indeed suggestive when applied to typical concepts in Statistics such as Fisher score and statistical entropy. The derivation of the Euler–Lagrange equations is classically done in the set-up of the Riemannian geometry, while here we have used the affine structure of Information Geometry. The present provisional results prompt a generalization to non-finite sample spaces and the development of applications. Finally, the related Hamiltonian formalism remains to be investigated.
